# Three-dimensional mitochondria reconstructions of murine cardiac muscle changes in size across aging

**DOI:** 10.1152/ajpheart.00202.2023

**Published:** 2023-08-25

**Authors:** Zer Vue, Kit Neikirk, Larry Vang, Edgar Garza-Lopez, Trace A. Christensen, Jianqiang Shao, Jacob Lam, Heather K. Beasley, Andrea G. Marshall, Amber Crabtree, Josephs Anudokem, Benjamin Rodriguez, Benjamin Kirk, Serif Bacevac, Taylor Barongan, Bryanna Shao, Dominique C. Stephens, Kinuthia Kabugi, Ho-Jin Koh, Alice Koh, Chantell S. Evans, Brittany Taylor, Anilkumar K. Reddy, Tyne Miller-Fleming, Ky’Era V. Actkins, Elma Zaganjor, Nastaran Daneshgar, Sandra A. Murray, Bret C. Mobley, Steven M. Damo, Jennifer A. Gaddy, Blake Riggs, Celestine Wanjalla, Annet Kirabo, Melanie McReynolds, Jose A. Gomez, Mark A. Phillips, Vernat Exil, Dao-Fu Dai, Antentor Hinton

**Affiliations:** ^1^Department of Molecular Physiology and Biophysics, Vanderbilt University, Nashville, Tennessee, United States; ^2^Department of Internal Medicine, University of Iowa, Iowa City, Iowa, United States; ^3^Microscopy and Cell Analysis Core Facility, Mayo Clinic, Rochester, Minnesota, United States; ^4^Central Microscopy Research Facility, University of Iowa, Iowa City, Iowa, United States; ^5^Department of Life and Physical Sciences, Fisk University, Nashville, Tennessee, United States; ^6^Department of Biological Sciences, Tennessee State University, Nashville, Tennessee, United States; ^7^Department of Cell Biology, Duke University School of Medicine, Durham, North Carolina, United States; ^8^J. Crayton Pruitt Family Department of Biomedical Engineering, University of Florida, Gainesville, Florida, United States; ^9^Department of Medicine, Baylor College of Medicine, Houston, Texas, United States; ^10^Division of Genetic Medicine, Department of Medicine, Vanderbilt University Medical Center, Nashville, Tennessee, United States; ^11^Department of Pathology, Carver College of Medicine, University of Iowa, Iowa City, Iowa, United States; ^12^Department of Cell Biology, School of Medicine, University of Pittsburgh, Pittsburgh, Pennsylvania, United States; ^13^Department of Pathology, Microbiology and Immunology, Vanderbilt University Medical Center, Nashville, Tennessee, United States; ^14^Department of Medicine, Vanderbilt University Medical Center, Nashville, Tennessee, United States; ^15^Tennessee Valley Healthcare Systems, United States Department of Veterans Affairs, Nashville, Tennessee, United States; ^16^Department of Biology at San Francisco State University, San Francisco, California, United States; ^17^Division of Clinical Pharmacology, Department of Medicine, Vanderbilt University Medical Center, Nashville, Tennessee, United States; ^18^Department of Biochemistry and Molecular Biology, Pennsylvania State University, State College, Pennsylvania, United States; ^19^Department of Medicine, Vanderbilt University Medical Center, Nashville, Tennessee, United States; ^20^Department of Integrative Biology, Oregon State University, Corvallis, Oregon, United States; ^21^Division of Cardiology, Department of Pediatrics, St. Louis University School of Medicine, St. Louis, Missouri, United States; ^22^Department of Pediatrics, Carver College of Medicine, University of Iowa, Iowa City, Iowa, United States; ^23^Department of Pathology, Johns Hopkins University School of Medicine, Baltimore, Maryland, United States

**Keywords:** aging, cardiac muscle mitochondria, MICOS, serial block-face SEM, three-dimensional morphometry

## Abstract

With sparse treatment options, cardiac disease remains a significant cause of death among humans. As a person ages, mitochondria breakdown and the heart becomes less efficient. Heart failure is linked to many mitochondria-associated processes, including endoplasmic reticulum stress, mitochondrial bioenergetics, insulin signaling, autophagy, and oxidative stress. The roles of key mitochondrial complexes that dictate the ultrastructure, such as the mitochondrial contact site and cristae organizing system (MICOS), in aging cardiac muscle are poorly understood. To better understand the cause of age-related alteration in mitochondrial structure in cardiac muscle, we used transmission electron microscopy (TEM) and serial block facing-scanning electron microscopy (SBF-SEM) to quantitatively analyze the three-dimensional (3-D) networks in cardiac muscle samples of male mice at aging intervals of 3 mo, 1 yr, and 2 yr. Here, we present the loss of cristae morphology, the inner folds of the mitochondria, across age. In conjunction with this, the three-dimensional (3-D) volume of mitochondria decreased. These findings mimicked observed phenotypes in murine cardiac fibroblasts with CRISPR/Cas9 knockout of *Mitofilin, Chchd3, Chchd6* (some members of the MICOS complex), and *Opa1*, which showed poorer oxidative consumption rate and mitochondria with decreased mitochondrial length and volume. In combination, these data show the need to explore if loss of the MICOS complex in the heart may be involved in age-associated mitochondrial and cristae structural changes.

**NEW & NOTEWORTHY** This article shows how mitochondria in murine cardiac changes, importantly elucidating age-related changes. It also is the first to show that the MICOS complex may play a role in outer membrane mitochondrial structure.

## INTRODUCTION

The mitochondrion is an organelle that critically carries out oxidative phosphorylation in the cell and is implicated in many diseases, including Alzheimer’s disease, diabetes, and heart failure (1–3). Given the dependence on mitochondria to meet energetic demands, mitochondrial dysfunction and deterioration are associated with cell death, including autophagy and apoptosis (4–6). In addition to producing adenosine triphosphate (ATP), mitochondria also regulate cell signaling during various cellular processes, including calcium homeostasis, apoptosis, and immunity (5, 7–9). Interestingly, research also suggests that mitochondria play a significant role in aging (10, 11). Although the functions of mitochondria are well understood, the relationship between mitochondrial dysfunction and age-associated diseases requires further investigation.

Mitochondrial morphology is associated with mitochondrial bioenergetic capacity. Mitochondrial dynamics, mainly fusion and fission, provide a means for mitochondria to readily adapt to the specific energy demands of the cell. Fusion and fission are regulated by optic atrophy 1 (OPA1) and dynamin-related protein-1 (DRP1), respectively (4, 12–14). Although the loss of DRP1 results in an abundance of overly elongated and fused mitochondria (5), the loss of OPA1 gives rise to fragmented mitochondria (12, 15). Mitochondria may go through several forms in coordinated cycles of fusion and fission. If OPA1 and mitofusions become upregulated, fusion rates increase producing long, tubular mitochondria with more complex network formation (16). Under normal physiological conditions, tubular mitochondria function well. However, under conditions of stress, mitochondria have been shown to alter their formation into donut, blob, or fragmented shapes, the latter of which is caused by a high fission–fusion ratio (16–18). Importantly, the evaluation of mitochondrial structure is vital as deviations from typical tubular mitochondrial shape indicate lower ATP production and increased susceptibility to autophagosome degradation (19). Moreover, the loss of several mitochondrial genes has been implicated in cristae structure alterations (13, 20).

Cristae, the folds of the mitochondria inner membrane, house electron transfer chain complexes responsible for establishing the proton motive force, which ultimately produces ATP (21). The morphological arrangement of cristae ultimately determines the energetic capacity of mitochondria and has been implicated in playing a role in other functions including apoptosis and homeostasis (21). The mitochondrial contact site and cristae organizing system (MICOS) maintains the morphology of cristae (22). Similar to OPA1, the MICOS complex governs the dynamics of cristae structure (20, 22–24). The MICOS complex is also involved in the maintenance of cristae junctions, which are subcompartments for metabolites (25). Given the significance of cristae junctions, cristae may also be involved in other processes such as regulating calcium homeostasis and molecular signaling. In consideration of the significance of mitochondrial and cristae morphology in normal function, dysfunction of the MICOS complex could negatively impact health (24). However, the role of the MICOS complex in mitochondrial health during cardiac aging has yet to be defined. Given the understanding that mitochondria are implicated in the aging process (11), we hypothesized that changes in the MICOS complex, which is critical to maintaining cristae morphology (26, 27), may lead to age-associated changes in mitochondria.

Specifically, we sought to understand the role of the MICOS complex in cardiac muscle mitochondria as it may provide novel insights into maintaining normal mitochondrial health in the heart. This study combines an in vivo and in vitro approach that looks at cardiac muscle in mice, cardiac fibroblasts, and human induced pluripotent stem cell-derived cardiomyocytes (iPSC-CMs). Previously, mitochondrial-focused aging studies have focused on cardiomyocytes (28); however, these studies overlooked the MICOS complex in relation to the aging of the cardiac fibroblasts. Cardiac health is linked to insulin resistance and diabetes (1, 29). Heart failure is also associated with other mitochondrial processes (6, 30–32) such as endoplasmic reticulum stress, mitochondrial bioenergetics, insulin signaling, autophagy, and oxidative stress (33). Mitochondria have specialized types and structures associated with their roles in glycolysis and oxidative metabolism in cardiac fibroblasts (17, 34–36). While young mitochondria show extensive specialization, it is unclear whether this is also true for aged cardiac murine mitochondria, and there is little information quantitively analyzing how aging affects cristae and three-dimensional (3-D) mitochondrial structure across aging in cardiac tissue. In cardiac muscle, research has shown that across aging, mitochondria in between myofibrils, called intermyofibrillar mitochondria, have the most significant decrease in mitochondrial oxidative respiration function (37). However, the full extent of causes of these changes in the oxidative phosphorylation of intermyofibrillar mitochondria have yet to be elucidated. Given that heart failure risk increases with aging (38), it is vital to consider the role of mitochondrial dysfunction in the loss of heart function (33).

To better understand the relationship between cardiac mitochondrial structure and the aging process, we used transmission electron microscopy (TEM) and fluorescence for two-dimensional (2-D) micrographs to observe mitochondria and cristae. Different muscle types have distinct mitochondrial function (17, 34). However, it is unclear how these unique functioning differences change over age. To further understand how aging affected mitochondrial morphology, we looked at cristae morphology at three time points; 3 mo, 1 yr, and 2 yr, which represent “young,” “middle-aged,” and “old,” mice, respectively. We expanded this to include serial block-face scanning electron microscopy (SBF-SEM) with manual contour tracing to reconstruct 3-D mitochondrial morphology and structure. We then quantitatively analyzed the 3-D networks in mouse cardiac muscle samples at different age intervals. Notably, 3-D reconstruction is an important avenue as the 3-D morphology of mitochondria has been linked to their functional capacity (17). Using SBF-SEM, we were able to look at single organelles to compare the shape, morphology, count, complexity, and branching across different ages. Furthermore, we compared the aging effects to the loss of the MICOS complex in relation to mitochondrial morphology alterations. Specifically, we used CRISPR/Cas9 on cardiac fibroblasts to knockout (KO) three genes of the MICOS complex: *Chchd3* (Mic19), *Chchd6* (Mic25), and *Mitofilin* (Mic60), as well as *Opa1*, as a positive control, to see how the loss of the MICOS complex affected mitochondrial size, morphology, and oxygen consumption rate. Finally, we sought to understand the role of the MICOS complex, specifically *Chchd6*, in modulating the reactive oxygen species response in iPSC-CMs.

## EXPERIMENTAL PROCEDURES

### Animal Care and Maintenance

These protocols are previously described (14), and animals used in this study were cared for using standard procedures approved by The University of Iowa Animal Care and Use Committee (IACUC) that follow the recommendations of the National Institute of Health’s *Guide for the Care and Use of Laboratory Animals*. Wild-type (WT) male C57Bl/6J mice were exclusively used in experiments. They were housed at 22°C on a 12-h:12-h light/dark. They had free access to water and standard chow. Mice were grown to various ages as described in the main text. A mixture of 5% isoflurane and 95% oxygen was used to anesthetize mice.

### RNA Extraction and RT-qPCR

RNA was extracted from tissue using TRIzol (Invitrogen) and RNeasy kit (Qiagen Inc). The subsequent concentration of isolated RNA samples was measured using a NanoDrop 1000 (NanoDrop products, Wilmington, DE) spectrophotometer at an absorbance of 260 nm and 280 nm. Reverse transcription was conducted on isolated RNA (∼1 µg) using a High-Capacity cDNA Reverse Transcription Kit (Applied Biosciences, Carlsbad, CA). SYBR Green (Life Technologies, Carlsbad, CA) (1) was used for real-time quantitative PCR (qPCR). Three samples for each qPCR (∼50 ng) were placed in a 384-well plate that subsequently underwent qPCR in the ABI Prism 7900HT instrument (Applied Biosystems) (Table 1) (14). The following qPCR conditions were used: 1 cycle at 95°C for 10 min; 40 cycles of 95°C for 15 s; 59°C for 15 s, 72°C for 30 s, and 78°C for 10 s; 1 cycle at 95°C for 15 s; 1 cycle of 60°C for 15 s; and 1 cycle of 95°C for 15 s. The results were normalized to glyceraldehyde-3-phosphate dehydrogenase (GAPDH). Data are shown as fold changes.

**Table 1.** qPCR primers used

Gene	Primers	Sequence
*Opa1*	Forward	5′- ACCAGGAGACTGTGTCAA-3′
	Reverse	5′- TCTTCAAATAAACGCAGAGGTG-3′
*Chchd3*	Forward	5′- GAAAAGAATCCAGGCCCTTCCACGCGC-3′
	Reverse	5′- CAGTGCCTAGCACTTGGCACAACCAGGAA-3′
*Chchd6*	Forward	5′- CTCAGCATGGACCTGGTAGGCACTGGGC-3′
	Reverse	5′- GCCTCAATTCCCACATGGAGAAAGTGGC-3′
*Mitofilin*	Forward	5′- CCTCCGGCAGTGTTCACCTAGTAACCCCTT-3′
	Reverse	5′- TCGCCCGTCGACCTTCAGCACTGAAAACCTAT-3′

qPCR, quantitative polymerase chain reaction.

### CRISPR-Cas9 KOs

Primary mouse fibroblasts were isolated as previously described (39, 40). Briefly, after cervical dislocation of 8- to 12-wk-old mice, via sternotomy, the heart was exposed and transferred to 15-mL PBS centrifuge tube and incubated at 37°C. Aorta was removed, and heart was washed with 10 mL of PBS warmed to 37°C. Once ventricles were dissected, they were minced into small pieces of 1 mm^3^, which were then subjected to enzymatic digestion using a mixture of collagenase-dispase and DNase I in PBS, while the tissues were incubated at 37°C with 5% CO_2_ atmosphere for 10 min on a rocking platform. After being centrifuged for 5 min at 1,500 rpm, the tissue digest pellet was resuspended in 1 mL of fibroblast culture medium (M199 medium supplemented with 10% FBS and 2% penicillin-streptomycin). This process was repeated approximately nine times until the tissue was completely digested. Individual cell suspensions were combined and pelleted again by running in a centrifuge at 1,500 rpm for 7 min. Cells were resuspended in cardiac fibroblast media and plated on 35-mm collagen-coated soft hydrogel-bound polystyrene plates. After the media is aspirated, plates with adherent cells were washed after 150 min with fibroblast culture medium. From there, 2 mL of fresh media was added, and the plate was incubated at 37°C with 5% CO_2_ atmosphere. After 20 h of digestion, this washing and replacing of media process was repeated, and cells were grown to 90% confluency.

Once isolated, CRISPR/Cas9 was used to infect cardiac fibroblasts to produce the following KOs: control CRISPR/Cas9 (sc-418922), *Chchd6* (Mic25) CRISPR (sc-425817), *Chchd3* (Mic19) CRISPR (sc-425804), and *Mitofilin* (Mic60) CRISPR (sc-429376) (Santa Cruz Biotechnology) (Table 2). For each CRISPR, 2.5% of the CRISPR was combined with 2.5% RNAiMax (Thermo Fisher Scientific; Cat. No. 13778075), and 95% Opti-MEM (Gibco; Cat. No. 31985070). This mixture was incubated at room temperature for 20 min. The media was removed from the cells and washing occurred twice with PBS. CRISPR mixture (200 µL) and Opti-MEM (800 μL) were added to each sample and then incubated at 37°C for 4 h. An additional 1 mL of DMEM was added before cells were incubated at 37°C overnight. Fibroblasts were washed with PBS, and a fresh medium was added. Experiments were performed 3 and 7 days following knockouts.

**Table 2.** RNA guide and plasmids used

Gene Name	Type of Plasmid	CAS Number
*Mitofilin*	CRISPR/Cas9 KO (m)	sc-429376
*Chchd6*	CRISPR/Cas9 KO (m)	sc-425817
*Chchd3*	CRISPR/Cas9 KO (m)	sc-425804
*Control*	CRISPR/Cas9 KO (m)	sc-418922

KO, knockout.

### Processing of Mouse Left Ventricles for Serial Block-Face Scanning Electron Microscope

Serial block-face scanning electron microscope (SBF-SEM) was performed as previously described (15). Male mice were euthanized using 5% isoflurane. The heart was excised and incubated in 2% glutaraldehyde with 100 mM phosphate buffer for 30 min. Left ventricles were dissected, cut into 1-mm^3^ cubes, and then incubated in 2.5% glutaraldehyde, 1% paraformaldehyde, and 120 mM sodium cacodylate solution for 1 h.

From there, the tissue was three times washed with 100 mM cacodylate buffer at room temperature. The tissue was immersed in 3% potassium ferrocyanide and 2% osmium tetroxide for 1 h at 4°C, then washed three times with deionized water and incubated in 0.1% thiocarbohydrazide and 2% filtered osmium tetroxide 30 min. Finally, the tissue was washed three times with deionized water, before transferring to 1% uranyl acetate, and left overnight at 4°C. The next day, the samples were washed with deionized water and then incubated at 0.6% lead aspartate solution for 30 min at 60°C. From there, the samples were dehydrated using an acetone gradient (20, 50, 70, 90, 95, and 100% acetone for 5 min each). Cardiac tissues were impregnated in Epoxy Taab 812 hard resin and then moved to new resin; polymerization occurred at 60°C for 36–48 h. Blocks of resin were sectioned, cut to 0.5 mm × 0.5 mm, and glued to aluminum pins. These pins were transferred to the FEI/Thermo Scientific Volumescope 2 SE Serial sections (300−400 ultrathin; 0.09 µm) from each block were collected for conventional TEM. All sections were collected onto formvar-coated slot grids (Pella, Redding CA), stained, and imaged.

### Measurement of OCR Using Seahorse

Oxygen consumption rate was measured for *Opa1*, *Cchchd3*, *Chchd6*, or *Mitofilin* KD fibroblasts using an XF24 bioanalyzer (Seahorse Bioscience: North Billerica, MA), as previously described (14, 41). Fibroblasts were plated at a density of 20 × 10^3^ per well and differentiated for 3 days. For KO models, CRISPR/Cas9 for the relevant KO was added as per the protocol above before plating. The media was changed to XF‐DMEM (supplemented with 1 g/L d-glucose, 0.11 g/L sodium pyruvate, and 4 mM l-glutamine), and cells were incubated without CO_2_ for 60 min. Cells were treated with oligomycin (1 μg/mL), carbonyl cyanide 4-(trifluoromethoxy)phenylhydrazone (FCCP; 1 μM), rotenone (1 μM), and antimycin A [10 μM (Fig. 5*I*)], in that order, while remaining in the XF-DMEM media. After measurement, cells were lysed accordingly to prior protocols (41), using 20 μL of 10 mM Tris with 0.1% Triton X-100 added at pH 7.4, and media replaced with 480 μL of Bradford reagent. Total protein concentration was then measured at absorbance at 595 nm and used for normalization. For each sample, three independent experiments were performed for each condition with representative data from the replicates being shown.

### Quantification of TEM Micrographs and Parameters Using ImageJ

The National Institutes of Health (NIH) ImageJ software was used to quantify TEM images, as previously described (42). Specifically, to ensure no bias in TEM quantification, one team member was responsible for conducting the experiment and fixing the cells and tissue, while another individual was responsible for processing and acquiring images using the electron microscope in a blinded and randomized manner. Each fiber of interest was divided into four equal quadrants, and two quadrants were randomly selected for measurements. Three additional blinded members were responsible for quantifying the anonymized samples. By averaging their collective findings, the potential for individual subjective bias was reduced. Moreover, those in charge of quantification were given randomized images at both whole cell and higher magnification levels to further minimize bias.

### Segmentation and Quantification of 3-D SBF-SEM Images Using Amira

SBF-SEM images were manually segmented in Amira to perform 3-D reconstruction, as previously described (15). For each 3-D reconstruction, (300−400 slices) were obtained and transferred to Amira. By hand, an individual blinded to samples traced structural features manually on sequential slices of micrograph blocks. For each of the 3-D reconstructions of cardiac muscle in mice, 50–100 serial sections were chosen at approximately equal *z*-direction intervals, stacked, aligned, and visualized using Amira to make videos and quantify volumetric structures. Algorithms for measurements were entered manually for those not already in the system (algorithms used are displayed in the main text; Fig. 3, *D* and *H*). A total of 750 mitochondria from three mice were collected for each quantification.

### Confocal mCherry-Mito-7 Labeling

To label the mitochondria of cardiac fibroblasts, the mCherry-Mito-7 plasmid was transfected into the cells using a transfection reagent according to the manufacturer’s instructions (43). Briefly, the plasmid and the transfection reagent were diluted in Opti-MEM medium separately, mixed gently, and incubated for 20 min at room temperature. The mixture was added to the culture medium of the cells, and the cells were incubated for 24–48 h to allow expression of the mCherry-Mito-7 protein. The localization of the mCherry-Mito-7 protein in cardiac fibroblasts was visualized using a Leica SP8 Confocal Microscope. The cells were washed with PBS, fixed with 4% paraformaldehyde for 10 min, and mounted with DAPI-containing mounting medium. The fluorescent signals were observed using appropriate filters and recorded using a digital camera.

### Immunostaining Protocol

For immunostaining, the hiPS-CMs were plated on glass-bottom dishes and were fixed in 4% paraformaldehyde for 15 min, permeabilized with 0.1% Triton-X-100 for 8 min, and blocked with normal donkey serum for 1 h, followed by incubation with primary antibody, Cardiac troponin-T (Invitrogen, Cat. No. MA5-12960) overnight. They were then probed with Donkey anti-mouse AlexaFluor 555 secondary antibody (Thermo) and counterstained with DAPI. Images were acquired using a Leica SP8 confocal microscope followed by quantification using ImageJ software.

### Segmentation and Quantification of 3-D Fibroblasts Using Confocal Microscopy and Imaris

For the 3-D reconstruction of fibroblasts, a minimum of 10 cells were chosen, and approximately 20 mitochondria from each cell were segmented for a total of about 200 mitochondria. 3-D structures were quantified as previously described (15) using the Imaris software (Bitplane), which automatically measured many parameters. All individuals measuring the parameters were blind to the experimental conditions but familiar in identifying diverse mitochondrial morphology. For each of the 3-D reconstructions of cardiac fibroblasts, 50–100 serial sections were chosen at approximately equal *z*-direction intervals, stacked, aligned, and visualized.

### Maintenance of Induced Pluripotent Stem Cell-Derived Cardiomyocytes

Cultured on Matrigel‐coated plates (Corning, Life Sciences), iPSC was fed 75% SFM and 25% mTeSR^+^. Cardiomyocyte differentiation was performed according to prior procedures (44), using STEMdiff Cardiomyocyte Differentiation and Maintenance kits. Typically, CMs began beating at *day 8*, and were further enriched in *day 10* of differentiation with lactic acid (sodium l‐lactate, Sigma-Aldrich). To aid CM maturation, triiodothyronine (T3) was added across *days 20*–*40*.

### Live-Cell Staining

Live cell staining procedures for iPS‐CMs were performed according to prior protocols (44). Once plated on glass‐bottom dishes, iPS-CMs media were replaced with 30 min with culture medium (37°C) supplemented with DCFDA (5 mM) and TMRE (25 nM). Media was replaced with new media supplemented with Hoechst 33342 for counterstaining. A Leica SP8 confocal microscope was used for image acquisition and ImageJ software was used for the analysis of fluorescence intensity.

### MitoSox

A stock solution of MitoSOXTM Red CMXRos-M7512 at a concentration of 1 mM was prepared by adding one vial of MitoSOXTM Red (50 µg) to 127 µL of DMSO, which was diluted to a working solution of MitoSOXTM Red CMXRos at a concentration of 2.5 µM by adding 10 µL of the stock solution to 10 mL of media. Cells were first aspirated of their media and then incubated with the working solution of MitoSOXTM Red at 37°C for 35 min. Following rinsing with warm media, cells were fixed with 16% paraformaldehyde at 37°C for 15 min. Following fixation, the cells were rinsed and imaged at 579-nm excitation and 599-nm emission wavelengths. Quantification of relative intensity normalized to standardized image area.

### Echocardiographic Parameters Collection

Animals were studied in accordance with protocols approved by the Institutional Animal Care and Use Committees of the Carver College of Medicine of the University of Iowa. Data from 9–10 animals at each age cohort were averaged to study in vivo cardiac structure and function per previous procedures (45, 46). After anesthetization in an induction chamber using 2.5% isoflurane, mice were placed on a heated electrocardiography platform for heart rate monitoring during the imaging procedure and maintained at 37°C. Mice had a nose cone administering 1% isoflurane while in the left lateral decubitus position during imaging. A 13-MHz probe (Vivid V echocardiograph; GE Healthcare, Tampa, FL) was used to take standard B-mode M-mode images, along the short-axis position of the papillary muscles.

### Data Analysis

All SBF-SEM and TEM data were obtained from at least three independent experiments and are presented as the means across these experiments. In the presentation, black bars represent the standard error of means, and dots represent individual data points. For all data with only two groups, an unpaired *t* test was used. If more than two groups were compared, one-way ANOVA was performed, and significance was assessed using Tukey’s post hoc tests for multiple comparisons. For both analyses, GraphPad Prism software package was used (La Jolla, CA). A minimum threshold of *P* < 0.05 indicated a significant difference. Higher degrees of statistical significance were defined as *P* < 0.01, *P* < 0.001, and *P* < 0.0001. For genetically regulated gene expression, statistical analysis was performed as previously described (47). Briefly, transcriptome-wide association studies using gene, splicing, and proteome were compared with Bonferroni correction (0.05/number of genes tested) for multiple testing correction. For all mouse models (Figs. 2 and 3), each dot represents an independent mouse (*n* = 3), but the statistical analysis shown represents the 750 mitochondria quantified.

## RESULTS

### Ultrastructural Changes in Mitochondria and Cristae with Age

It is commonly understood that heart function declines with age in human and murine models. However, these changes and their association with mitochondrial morphological changes are unclear. To investigate the cardiac function of aged mice cohorts, before TEM analysis, we collected and analyzed echometric data. We observed changes with age that included increased cardiac output, stroke volume, and LV thickness, without significant changes in normalized heart mass, ejection fraction, or heart rate (Supplemental Fig. S1: https://doi.org/10.6084/m9.figshare.22861616.v1).

As expected, mice increased in body weight across aging (Supplemental Fig. S2A). Specifically, the fat mass increased significantly faster than lean mass, especially in the 2-yr sample (Supplemental Fig. S2, *B* and *C*). In accordance with this, heart mass also significantly increased with age (Supplemental Fig. S2*D*). After a 6-h fast, a glucose tolerance test (GTT) showed that aged mice had higher plasma glucose levels post-GTT, indicating impaired glucose tolerance (Supplemental Fig. S2, *E* and *F*). These results are consistent with aging mice having larger fat depots and abnormal glucose status in comparison to younger mice. Critically, on average, even with decreased glucose tolerance, aged mice did not reach blood glucose levels that are indicative of diabetes. Therefore, this cohort served as a strong representation of age-independent from pathology in this study. To further understand how mitochondrial affected these dynamics, TEM was used to understand aging mitochondrial and cristae morphology.

**Figure 1.**
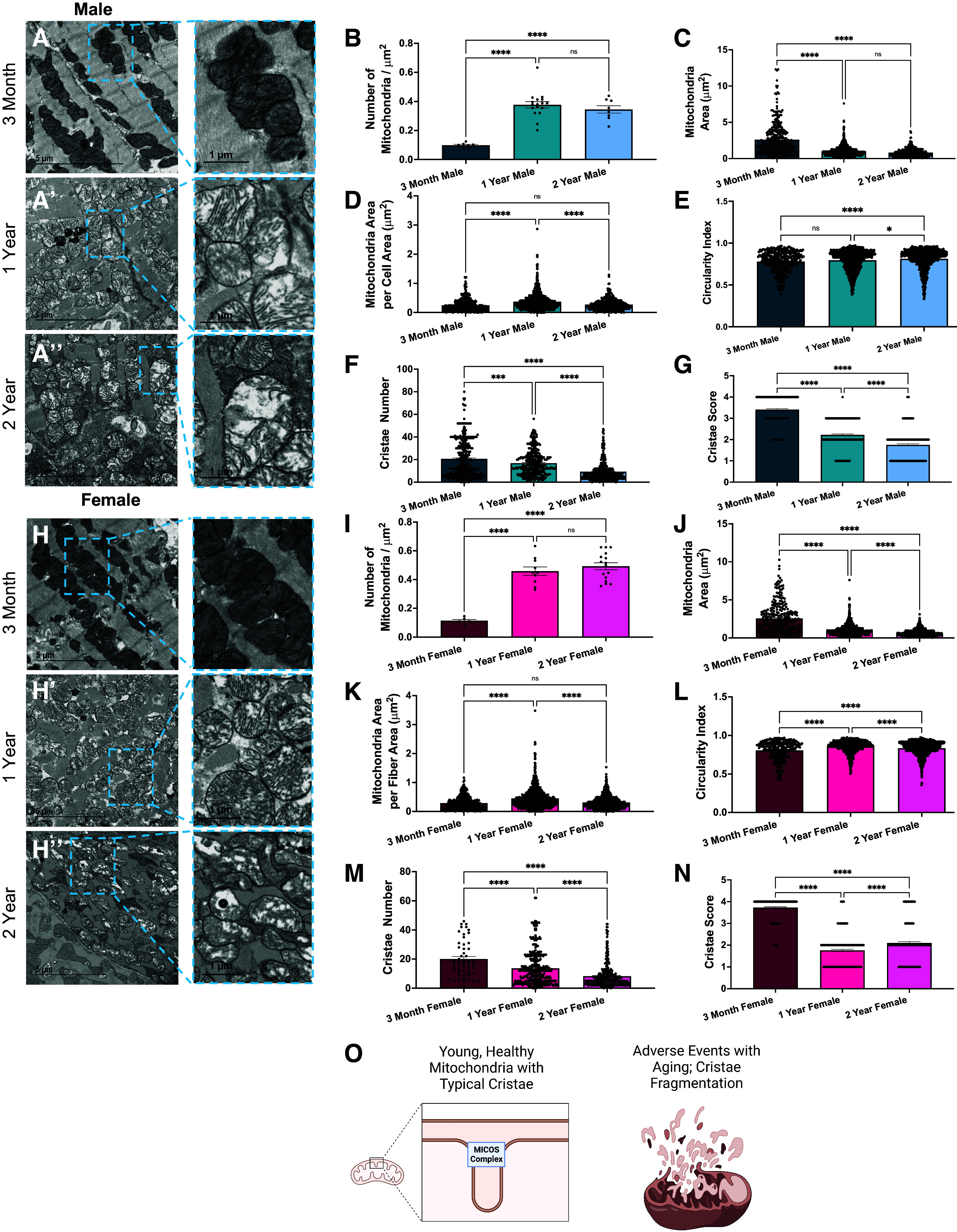
Changes in cardiac muscle mitochondria and cristae across aging revealed in transmission electron microscopy (TEM) and heart data. Representative transmission electron micrographs for cardiac muscle at 3 mo (*A*), 1 yr (*A′*), and 2 yr (*A′′*) in male mice. Blue boxes show cristae magnified to enhance the details of cristae. Quantification of key mitochondrial characteristics included number of mitochondria normalized to area surveyed (*B*), average mitochondrial area (*C*), total mitochondrial area content per fiber area (*D*), and circularity index (*E*) that measures mitochondrial shape. For cristae, number of cristae (*F*) and cristae score (*G*), a measurement of the quality of cristae observed, are shown. Representative transmission electron micrographs for cardiac muscle at 3 mo (*H*), 1 yr (*H′*), and 2 yr (*H′′*) in female mice. Quantification of number of mitochondria normalized to area surveyed (*I*), average mitochondrial area (*J*), mitochondrial area per fiber area (*K*), circularity index (*L*), number of cristae (*M*), and cristae score (*N*). *O*: schematic showing dysfunction of mitochondrial cristae across the aging process. Each dot represents a single mitochondrion. MICOS, mitochondrial contact site and cristae organizing system. One-way ANOVA statistical test was performed with post hoc Tukey’s test. Significance values indicate *P ≤ 0.05, **P ≤ 0.01, ***P ≤ 0.001, and ****P ≤ 0.0001; ns, not significant. Images were created using a licensed version of BioRender.com.

We examined mitochondria and cristae morphology in cardiac muscle at 3-mo-, 1-yr-, and 2-yr-old male mice. Different cell types can be studied in cardiac muscle (36) and cardiac muscle can give information about potential causes of loss of optimal heart function with age (38). TEM is a powerful tool for studying cristae in mitochondria as it gives very high-quality micrographs (42). Young male mice (*n* = 3, per age group) showed very clear mitochondria with electron-dense membranes, whereas the aged mice had fewer mitochondria and cristae (Fig. 1, *A–A′′*). The number of mitochondria more than quadrupled between the 3-mo to 1-yr aged samples before slightly leveling out between 1 and 2 yr (Fig. 1*B*). Although mitochondrial abundance increased, the average mitochondrial area was significantly smaller in samples older than 3-mo; the decrease is smaller between the 1- and 2-yr samples (Fig. 1*C*). This shows a compensating effect as mitochondrial area per fiber area does not show significant changes across the total aging process (Fig. 1*D*). The mitochondria also became slightly more circular (Fig. 1*E*). To measure ultrastructural details of mitochondria, cristae numbers in each mitochondrion were evaluated and consistently showed that the number of cristae decreased with age (Fig. 1*F*). Finally, the cristae score was used to assess the cristae quantity and morphology as it is often used to holistically evaluate cristae (42, 48). A cristae score of 0 represents no clearly defined cristae, 1 represents that greater than 50% of mitochondrial area is devoid of cristae, a cristae score of 2 represents that less than 75% of the mitochondria area has cristae, and the maximum cristae score of 4 represents overall typically defined cristae with normal architecture. There was a large drop in the quality of cristae in cardiac muscle with age. The young 3-mo sample had mostly regular and slightly irregular cristae, with cristae scores of about 3.3. The aged samples both had cristae scores around 2, which suggests there are many areas lacking cristae or having irregular cristae (Fig. 1*G*). Thus, we found that cristae count and quality both decreased across the aging process. These important findings that indicate a loss of cristae folds with aging caused us to wonder if cristae were differentially affected in a female model (Fig. 1, *H–H′′*).

Previous research has indicated that male mice have impaired mitochondrial function in cardiac muscle in comparison to female mice, in both healthy and cardiac pathological mice (49). To see mitochondrial and cristae structure changes across aging in a sex-dependent manner, we first looked at mitochondrial quantity that increased after a year of aging, while mitochondrial area was inversely proportional (Fig. 1, *I–K*). Like the male model, the circularity index of mitochondria increased with age (Fig. 1*L*). Importantly, the female model recapitulated findings of significant loss of cristae across the aging process in murine cardiac tissue. Specifically, the number of cristae decreased (Fig. 1*M*) consistently across aging, while the cristae score also significantly decreased past the 3-mo age point (Fig. 1*N*). Although in the female model, the cristae score improved slightly in comparing 2-yr-old with 1-yr-old mice, in both cohorts the average cristae score remained below 2, representing that 25% or more of mitochondrial area lacked typical cristae. Based on these findings, in tandem, we propose that mitochondrial fission, resulting in decreased mitochondrial area, is increased while the quality of cristae is decreased with age in a non-sex-dependent manner (Fig. 1*O*). However, it should be noted that TEM can be limited in analyzing mitochondrial changes beyond those of cristae structure changes across the aging process. Given these changes did not present as sex dependent, to properly understand how potential novel phenotypes arose in cardiac tissue, alterations in mitochondrial morphology were analyzed with 3-D reconstruction in a male mouse model.

### Aging Changes Mitochondrial Size in Cardiac Muscle: 3-D Reconstruction Analysis

**Figure 2.**
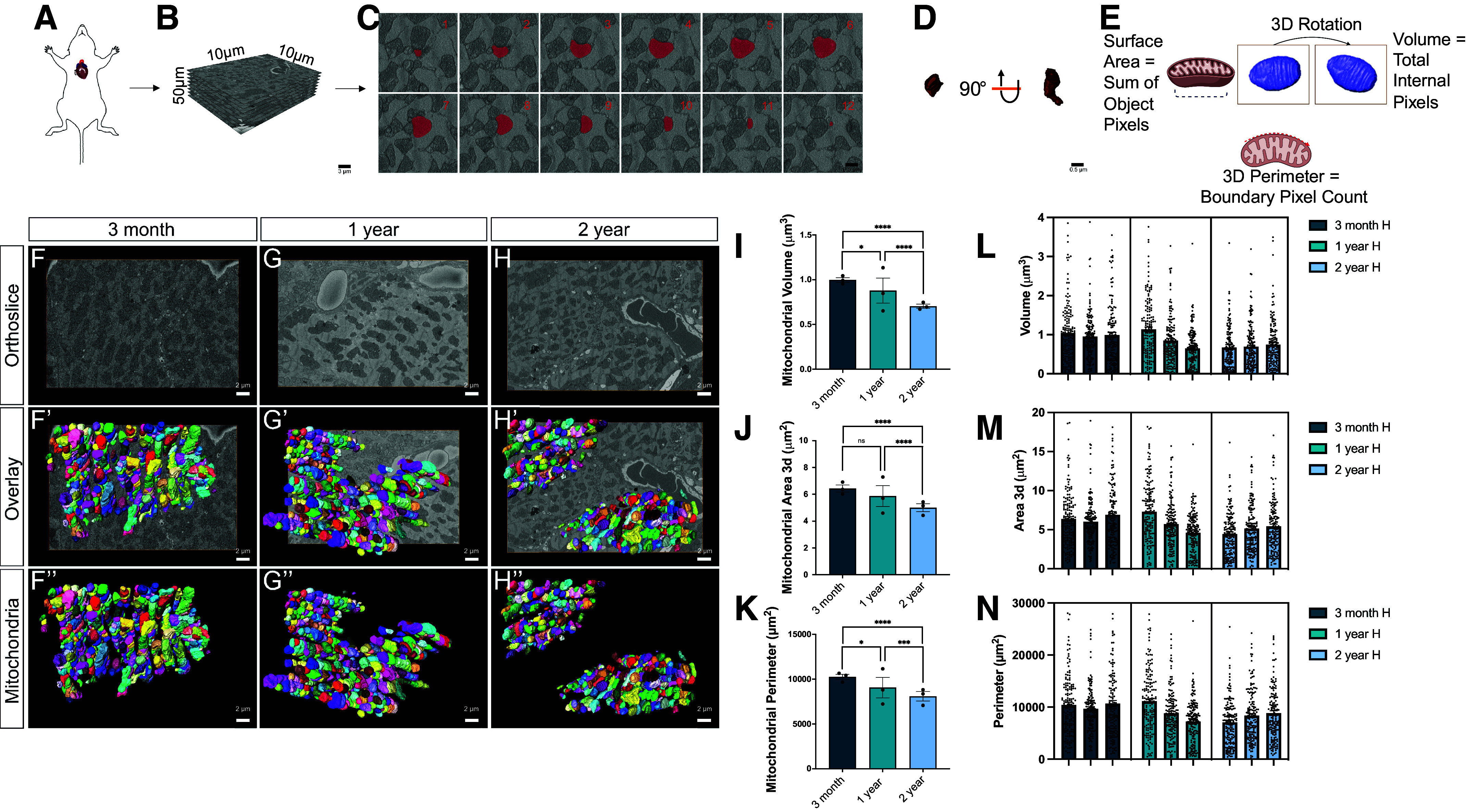
Changes in cardiac muscle mitochondria morphology across aging revealed in serial block facing-scanning electron microscopy (SBF-SEM). *A*: workflow depicting removal of the left ventricle of murine hearts. *B*: following embedded fixation, SBF-SEM allows for ortho slice alignment. *C* and *D*: manual segmentation of ortho slices was performed (*C*) to ultimately yield 3-dimensional (3-D) reconstructions of mitochondria (*D*). *E*: schematic depicting how metrics are found using preinstalled analyses on Amira 3-D Software. For some graphs, outlying dots were removed for presentation, but all mitochondria values were considered in statistical analysis. *F–H*: representative SBF-SEM orthoslice for cardiac muscle (*n* = 3 mice). *F′–H′*: 3-D reconstructions of mitochondria in male cardiac tissues of different ages overlaid on ortho slices. *F′′–H′′*: 3-D reconstructed and isolated mitochondria for clear visualization. *I–K*: 3-D reconstructions were then quantified by mitochondrial volume in cardiac muscle of different ages (*I*), 3-D area of the average mitochondria in cardiac muscle (*J*), and perimeter of the average mitochondria in cardiac muscle (*K*). Each dot represents the average of a single mouse. *L–M*: individual quantifications for average volume per mitochondria (*L*), average 3-D area of mitochondria (*M*), and average perimeter of the mitochondria (*N*) for each of the three mice sampled at 3 mo, 1 yr, and 2 yr. One-way ANOVA statistical test performed with post hoc Tukey’s test. Significance values indicate *P ≤ 0.05, ***P ≤ 0.001, and ****P ≤ 0.0001; ns, not significant. Images were created using a licensed version of BioRender.com.

Based on our observations of the lack of cristae folding in aging cardiac muscle, we used 3-D techniques to image cardiac muscle biopsies from young (3 mo old), mature (1 yr old), and aged (2 yr old) mice with SBF-SEM With ranges of 10 µm for the *x*- and *y*-planes and 50 µm for the *z*-plane, SBF-SEM enables 3-D reconstruction of mitochondria with an accurate spatial resolution that cannot be seen in 2-D. Given TEM did not reveal a sex-specific change, we focused on a male model. Specifically, we examined the morphological changes in the intermyofibrillar region as mitochondrial frequency has been demonstrated to increase in this region (50). Thus, there is a need to determine if such changes in frequency are associated with other mitochondrial-related aging changes, including mitochondrial orientation, network, and nanotunnel alterations. To elucidate the changes in intermyofibrillar mitochondria in relation to aging, we surveyed ∼250 intermyofibrillar mitochondria from each of the three male mice (*n* = 3) (Fig. 2*A*) sampled at each age time point with SBF-SEM 3-D reconstruction methods. At a 10 µm by 10 µm image stack resolution, ∼300 serial section orthoslices with a total imaging depth of 50 μM (Fig. 2*B*) were manually traced at transverse intervals (Fig. 2*C*). This allowed for 3-D reconstructions of mitochondria to be created, as observed in the flowchart of Fig. 2 (Fig. 2*D*). In totality, across the three mice, ∼750 mitochondria for each age cohort were surveyed. All measurements used 3-D metrics to understand the volumetric dynamics of mitochondria (Fig. 2*H*).

In Fig. 2, we show representative images of the cardiac muscle at each aging point (Fig. 2, *F–H*). The overlay of the 3-D reconstruction (Fig. 2, *F′–H′*), and the isolated 3-D reconstruction (Fig. 2, *F′′–H′′*), allow for the mitochondrial structure to be viewed better (Supplemental Videos S1 and S2). Each color represents an independent mitochondrion. The perimeter, 3-D area (or surface area), and volume decreased between 1 and 2 yr, which was a greater change than between 3 mo and 1 yr (Fig. 2, *I–K*). Importantly, this shows that while TEM was able to capture some dynamics of mitochondria, it neglected the alteration that is far greater in mice moving from adulthood to seniority (1 to 2 yr) than from adolescence to adulthood (3 mo to 1 yr). For these quantifications, three individual mice were sampled at each age (Fig. 2, *L–N*). When mitochondrial quantifications from each mouse were compared, they showed overall little intergroup heterogeneity but persistent intraindividual variability.

**Figure 3.**
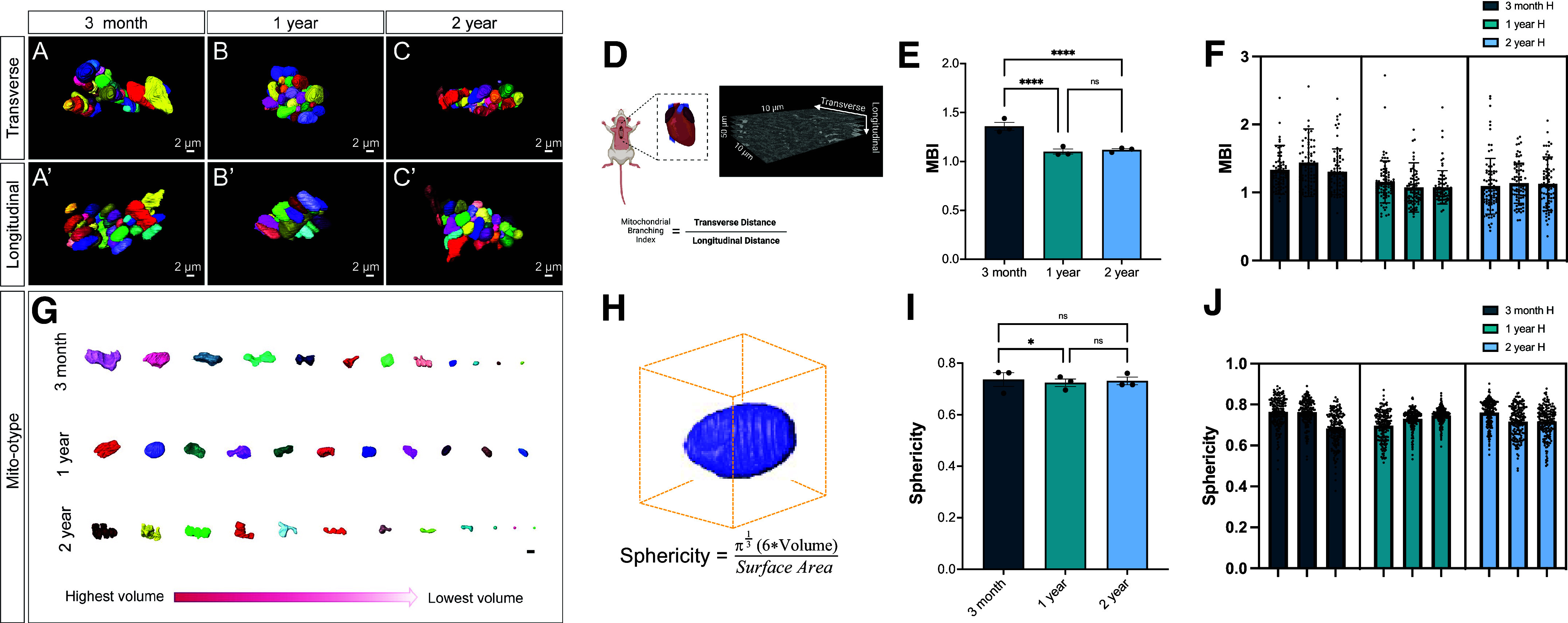
Changes in cardiac muscle branching and networking across aging revealed in serial block facing-scanning electron microscopy (SBF-SEM). *A–C*: three-dimensional (3-D) reconstruction of individually colored mitochondria from a transverse view for mouse cardiac muscle of different ages. *A′–C′*: 3-D reconstruction of individually colored mitochondria from a longitudinal view in cardiac muscle tissues of different ages. *D*: schematic showing how transverse and longitudinal mitochondrial length is used to measure mitochondrial branching index. *E*: mitochondrial branching index was measured to estimate mitochondrial networks. *F*: for each of the three male mice sampled at each aging time point, the mitochondrial branching index is shown. *G*: mitochondria 3-D reconstructions were further organized by volume for each of the age cohorts. *H*: schematic showing how sphericity is measured, as a function of volume to surface area ratio. *I*: to measure shape, sphericity changes across aging in cardiac muscle was further measured. *J*: average sphericity values from each of the approximately 75 mitochondria surveyed per mice is also shown. Outlying dots were removed for presentation for some graphs, but all mitochondria values were considered in statistical analysis. One-way ANOVA statistical test performed with post hoc Tukey’s test. Significance values indicate *P ≤ 0.05 and ****P ≤ 0.0001; ns, not significant. Images were created using a licensed version of BioRender.com.

With these observations, we sought to determine if mitochondrial networks changed in response to aging. To further elucidate age-related changes, we looked at cardiac muscle from both transverse (Fig. 3, *A–C*) and longitudinal views (Fig. 3, *A′–C′*). The mitochondrial branching index (MBI) was used to better understand mitochondrial branching (Fig. 3*D*). The MBI measures network complexity by examining the ratio between transverse and longitudinal mitochondria (36) and is similar to aspect ratio which measures the ratio between major and minor axis (51). With this technique, we demonstrated that mitochondrial branching decreased between 3 mo and 1 yr (Fig. 3*E*). Similarly, within an age cohort, there is no significant variation between mice, but there was a large amount of heterogeneity in mitochondrial samples in each mouse (Fig. 3*F*). To further characterize the mitochondrial types in each age cohort, we used mito-otyping, a method similar to karyotyping, to organize mitochondria based on their volumes to better visualize the overall mitochondrial diversity (Fig. 3*G*). This allows for comparison of the mitochondria across ages at each volume. We measured sphericity to understand how the surface area changed during the aging process (Fig. 3*H*). Sphericity generally showed minimal changes (Fig. 3*I*) while there was homogeneity across all nine mice measured (Fig. 3*J*). For all these metrics, some outliers were observed, which were omitted in the presentation for ease of view, but they were included in all statistical analyses (Supplemental Fig. S3). Critically, together, this approach revealed that there were few significant changes in morphology with only branching showing reductions. In combination, the aged cardiac muscle mitochondrial morphology resembled healthy mitochondria with a reduced size that lacks a phenotype or fragmentation (Fig. 3). Since the largest findings are loss in cristae structure and mitochondria size across aging, we sought to understand the underlying cause of these observed changes. Given the principal role of the MICOS complex in cristae formation and maintenance (27, 52), we investigated its role in cristae and mitochondrial remodeling across the aging process in cardiac muscle.

### Aging Changes in MICOS Complex in Fibroblasts: 3-D Reconstruction Analysis

**Figure 4.**
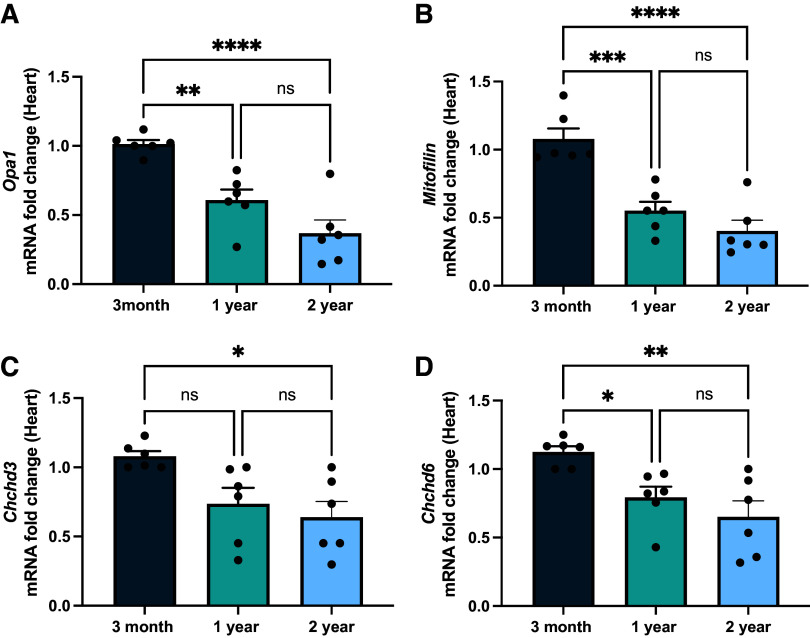
Transcription of *Opa-1* and mitochondrial contact site and cristae organizing system (MICOS) genes in aging cardiac muscle. *A–D*: quantitative polymerase chain reaction (qPCR) analyzing the gene transcript fold changes of Opa-1 and MICOS across aging: *Opa1* transcripts (*A*), *Mitofilin* transcripts (*B*), *Chchd3* transcripts (*C*), and *Chchd6* transcripts (*D*). One-way ANOVA statistical test performed with post hoc Tukey’s test. Significance values indicate *P ≤ 0.05, **P ≤ 0.01, ***P ≤ 0.001, and ****P ≤ 0.0001; ns, not significant. For all experiments, *n* = 6.

Although it is established that the MICOS complex is critical for mitochondrial dynamics (22, 26), it is unclear how aging affects the MICOS complex. Studies have shown that *Opa1*, which is epistatic to the MICOS complex and physically interacts with components of the MICOS complex (23), decreases with age (53). With *Opa1* as a positive control, we sought to determine if the MICOS complex mRNA expression also decreased in cardiac muscle with age. As previously suggested (53), *Opa1* mRNA decreased by over 50% between 3 mo and 2 yr (Fig. 4*A*). *Mitofilin* also decreased by 50% (Fig. 4*B*). Likewise, *Chchd3* and *Chchd6* also progressively decreased with age but not as much as the decline of other transcripts (Fig. 4, *C* and *D*). While *Opa1* interacts with the MICOS complex, it is not required for the formation of cristae junctions at which the MICOS complex forms, nor does *Opa1* loss negatively affect MICOS components (54). This suggests that the loss of the MICOS complex across aging occurs in an *Opa1*-independent manner.

**Figure 5.**
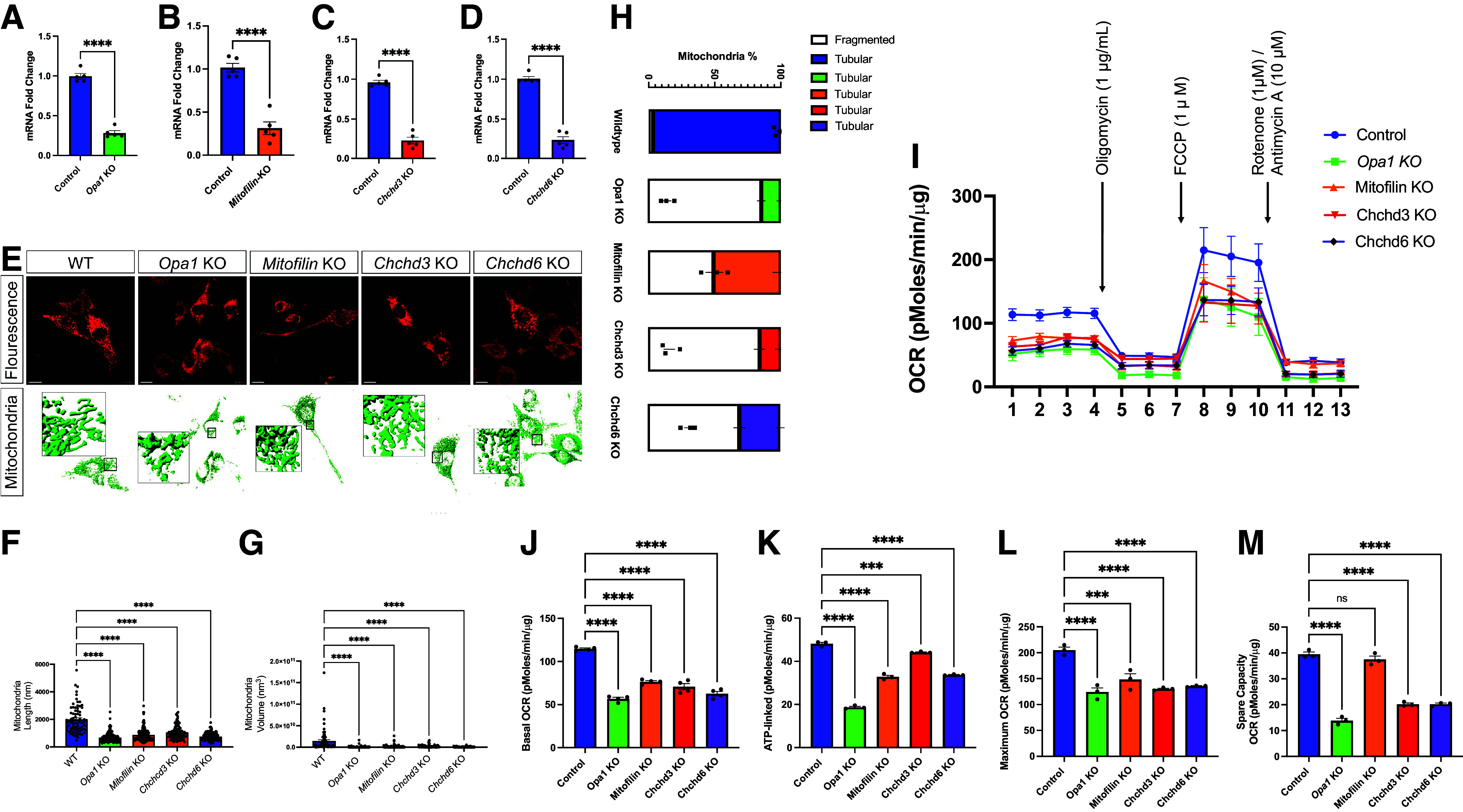
Loss of *Opa1* and mitochondrial contact site and cristae organizing system (MICOS) genes result in mitochondrial structure changes and oxygen consumption rate changes. *A–D*: quantitative polymerase chain reaction (qPCR) (*n* = 6) analyzing the gene transcript fold changes of Opa-1 and MICOS in cardiac fibroblasts upon CRISPR/Cas9 knockdown: *Opa1* transcripts (*A*), *Mitofilin* transcripts (*B*), *Chchd3* transcripts (*C*), and *Chchd6* transcripts (*D*). *E*: confocal fluorescence (using mCherry-Mito-7) shows changes upon individual knockout (KO) of *Opa1, Mitofilin, Chchd3,* and *Chchd6* in mouse cardiac muscle. Below this, individual KO of *Opa1, Mitofilin, Chchd3,* and *Chchd6* was also observed in 3-D reconstruction of SBF-SEM. *F–H*: quantification upon KO state of each MICOS gene and *Opa1* (*n* = 10 cells) was performed in 3-D reconstruction: mitochondria length (*F*) and mitochondria volume across each knockout (*G*); additionally, the relative frequency of mitochondria that presented as fragmented or tubular was altered across knock outs (*H*). *I*: Seahorse Analyzer was used to measure oxygen consumption rate (OCR) in *Opa1*, as positive control, along with *Chchd3*, *Chchd6,* and *Mitofilin* KD. *J–L*: basal OCR (*J*), which shows baseline energetic demands; ATP linked (*K*), which was measured by ATP-inhibitor oligomycin; maximum OCR (*L*), which represents maximum respiration capacity upon application of carbonyl cyanide 4-(trifluoromethoxy)phenylhydrazone (FCCP). Finally, application of rotenone/antimycin A causes only nonmitochondrial respiration to remain, which allows for measurement of spare respiratory capacity (*M*), which signifies the extra amount of ATP that can quickly be generated. For seahorse, *n* = 6 plates for experimental knockouts, while for control, *n* = 16. *A–D*: unpaired (independent) Student’s parametric statistical *t* test performed. For all other statistical tests, one-way ANOVA statistical test performed with Dunnett’s multiple comparisons test. Significance values indicate ***P ≤ 0.001 and ****P ≤ 0.0001; ns, not significant.

To further understand the role of mitochondrial dynamics upon the loss of these MICOS genes, we used CRISPR/Cas9 to make a KO model in fibroblasts, which was validated by qPCR (Fig. 5, *A–D*). We have previously validated DRP1, MFN2, and OPA1 CRISPR-mediated gene knockout translates to protein level changes as shown by transmission electron microscopy (42, 55). Coupled with the current use of qPCR, gene knockout translates to reduced transcript and protein expression. Specifically, we measured approximately 1,250 mitochondria across 10 cells. Since the loss of *Opa1* triggers changes in morphology (12, 15, 20, 42), we used it as a positive control for morphological changes. We marked mitochondria with MitoTracker Red and verified the successful deletion of *Opa1, Mitofilin, Chchd3,* and *Chchd6* (Fig. 5*E*). Then *z*-stacks were reconstructed using Bitplane Imaris to quantify the number of cardiac fibroblasts with loss of MICOS expression (Fig. 5*E*). Deletions of *Chchd3, Mitofilin, Chchd6,* or *Opa1* led to significant decreases in mitochondria length (Fig. 5*F*) and volume (Fig. 5*G*). We further sought to determine if the mitochondria were more tubular, which represents normal states, or fragmented, which represents stressed states (Fig. 5*H*). Notably, *Chchd3-*KO cells had nearly completely fragmented mitochondria, like those in *Opa1-*KO. Although the WT had mostly tubular mitochondria, the deletion of any of these critical mitochondrial proteins led to a much higher proportion of fragmented mitochondria. We further used live-confocal imaging to observe the dynamics of mitochondrial changes in real time. The WT mitochondria (Supplemental Video S3) were normal and potentially undergoing fusion. However, the cells with deletion of *Opa1* (Supplemental Video S4), *Chchd3* (Supplemental Video S5), or *Mitofilin* (Supplemental Video S6) showed fragmented mitochondria. These videos aid in showing the real-time mitochondrial dynamics that may be contributing to changes in the 3-D structure observed.

To understand how the loss of the MICOS complex affects mitochondrial activity, we measured oxygen consumption rates (OCRs) with an XF24 extracellular flux bioanalyzer upon knockout of *Opa1* and the MICOS genes in cardiac fibroblasts (Fig. 5*I*). The OCR measurements encompassed four stages: basal respiration, ATP-linked respiration, maximal respiratory capacity, and reserve capacity. Basal respiration reflects the cellular OCR under normal, nonstressed conditions, while ATP-linked respiration represents the OCR directly related to ATP synthesis, which can be measured following the application of oligomycin, an inhibitor of ATP synthase. Maximal respiratory capacity refers to the maximum OCR a cell can achieve under stress or high-energy demand and is measured following the application of carbonyl cyanide-*p*-trifluoromethoxyphenylhydrazone (FCCP), an uncoupler of mitochondrial oxidative phosphorylation. Reserve capacity represents the difference between maximal respiratory capacity and basal respiration, indicating the cell’s ability to respond to increased energy demand and is measured following the application of antimycin A/rotenone, inhibitors of the electron transport chain. The *Opa1*-KO, *Chchd3*-KO, *Chchd6*-KO, and *Mitofilin-*KO cells showed decreased basal OCR, ATP-linked OCR, and maximum OCR compared with the control (Fig. 5, *J–M*), indicating a general loss of OCR at baseline and during high demand.

These results suggest that the depletion of the MICOS complex can lead to the impairment of the electron transfer chain, mitochondrial respiration, and bioenergetics, providing valuable insights for the development of targeted therapies for diseases associated with mitochondrial dysfunction. Of relevance, while *Opa1*-KO had a dramatic decrease in the reserve capacity of OCR, *Mitofilin*-KO cells showed no alteration in reserve capacity. These data show decreased oxygen consumption rate at basal consumption, decreased ATP-linked respiration following oligomycin application, decreased maximal respiratory capacity following FCCP application, and decreased reserve capacity, as measured following antimycin A/rotenone treatment.

Loss of OPA1 does not yield changes in mtDNA (14), while alterations in the MICOS complex can affect mtDNA content (56, 57). Given these differential changes in mtDNA content, as shown previously, total protein is the most appropriate way to normalize to mitochondrial content (14, 58). Accordingly, cells were plated at a density of 20 × 10^3^ consistently with normalized protein, which together will consider changes from the mitochondrial mass. Together this shows that changes in oxidative consumption occur independently from changes in mitochondrial mass. Overall, depletion of these critical mitochondrial proteins led to a general loss of oxygen consumption rate both at baseline and during high demand. These findings suggest that depletion of the MICOS complex can lead to the impairment of the electron transfer chain along with mitochondrial respiration and bioenergetics.

### Loss of *Chchd6* Results in Changes in Mitochondrial Size, Shape, and Fluorescence of Reactive Oxygen Species in Induced Pluripotent Stem Cell-Derived Cardiomyocytes

**Figure 6.**
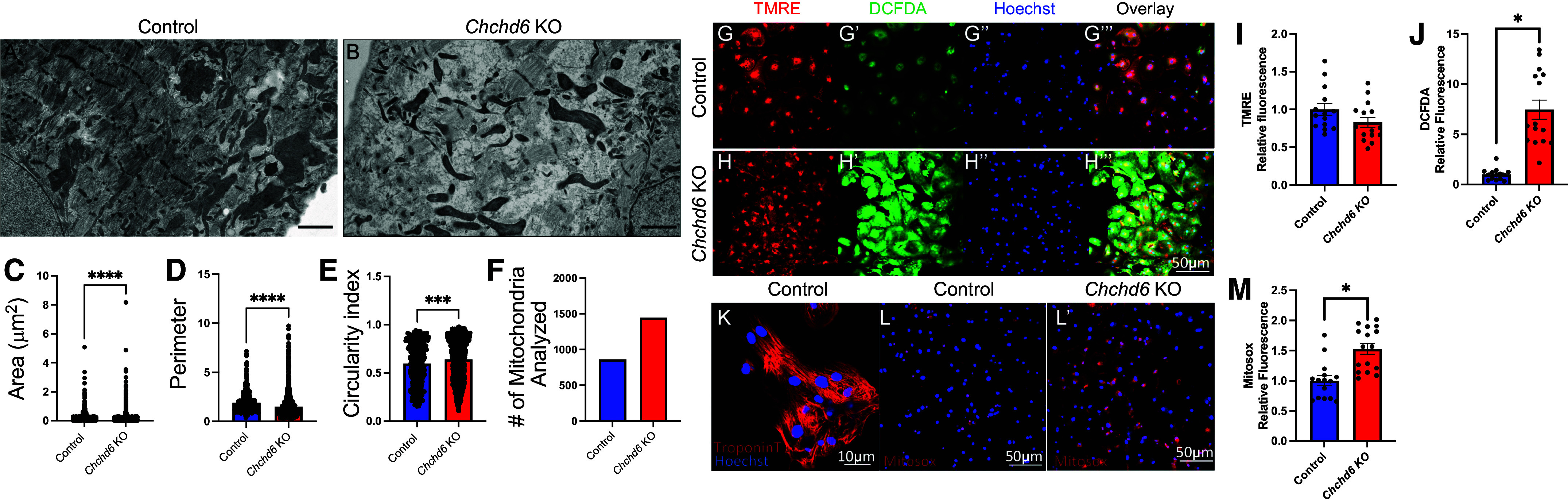
Chchd6-deficiency results in changes in mitochondrial amounts and reactive oxygen species in induced pluripotent stem cell-derived cardiomyocytes (iPSC-CMs). *A* and *B*: transmission electron microscopy (TEM) representative images of control (*A*) and CHCHD6-deficient induced pluripotent stem cell-derived cardiomyocytes (*B*). *C–F*: TEM analysis of area (*C*), perimeter (*D*), circularity index (*E*), and mitochondrial count (normalized to cell area) (*F*). Fluorescence live-cell imaging in control iPSC-CMs used tetramethylrhodamine, ethyl ester (TMRE; red) dye to label active mitochondria (*G*), dichlorodihydrofluorescein diacetate (DCFDA; green) to assay for overall reactive oxygen species (*G′*), and Hoechst dye (blue) to label for DNA (*G′′*). Overlaid fluorescence image is also presented (*G′′′*) and fluorescence dye analysis were further performed in Chchd6-deficient IPSC-SMs (*H–H′′′*). Analysis of fluorescent channels showing relative fluorescence of TMRE (*I*) and DCFDA (*J*). Further fluorescence dye with Hoechst for DNA and troponin-T (*K*) and MitoSox to measure reactive oxygen species including superoxides in control (*L*) and Chchd6-deficient IPSC-SMs (*L′*). *M*: analysis of MitoSox fluorescence. *C–F*: each dot represents a single mitochondrion. *I–M*: each dot represents quantification from a microscopic field from three independent replicates for each control and knockout (KO) groups. Unpaired (independent) Student’s parametric statistical *t* test performed. Significance values indicate *P ≤ 0.05, ***P ≤ 0.001, and ****P ≤ 0.0001; ns, not significant.

To understand the impact of the MICOS complex beyond murine cardiac fibroblast models, we knocked down *Chchd6*, a component of the MICOS complex, in induced pluripotent stem cell-derived cardiomyocytes (iPCS-CMs). To verify changes in mitochondrial size and morphology, we analyzed iPCS-CM through TEM (Fig. 6, *A* and *B*). We found that *Chchd6*-deficient iPCS-CM had reduced mitochondrial area and perimeter while mitochondrial circularity index increased (Fig. 6, *C–F*). We then moved to fluorescence live-cell imaging and overlaid tetramethylrhodamine, ethyl ester (TMRE) red dye, a label of active mitochondria and their membrane potential, dichlorodihydrofluorescein diacetate (DCFDA) green dye to assay for overall reactive oxygen species, and Hoechst blue dye to label for DNA in control iPCS-CMs (Fig. 6, *G–G′′′*) and *Chchd6*-depleted IPCS-CMs (Fig. 6, *H–H′′′*). Analysis showed that while mitochondrial membrane potential did not significantly change (Fig. 6*I*), general reactive oxygen species increased (Fig. 6*J*). Following verifying the cardiomyocyte identity of our iPSC-CM using troponin T staining (Fig. 6*K*), to further validate this response and to look at alterations in levels of superoxides (59) upon loss of the MICOS complex, we examined Mitosox live staining (Fig. 6, *L*–*L*′), which shows a significant increase of Mitosox fluorescence in the *Chchd6* depleted, suggesting that the loss of this subunit of the MICOS complex results in increased in mitochondrial superoxides.

We also sought to understand the functional impact of loss of Chchd6 using the Vanderbilt BioVU bank (Supplemental Fig. S4*A*). This is a powerful biobank of over 270,000 collected by the Vanderbilt University Medical Center (60). This database includes a mixture of individuals of European ancestry and African American ancestry (Supplemental Fig. S4*B*). Genetically regulated gene expression (GREX) of CHCHD6 was calculated across cardiac tissues in individuals of European ancestry. The relationship between CHCHD6 GREX and the “Heart failure with reduced EF [Systolic or combined heart failure]” phenotype was evaluated across cases and controls within BioVU. The associations between CHCHD6 GREX and heart failure were nominally significant in two of the three tissues tested in individuals of European ancestry and one of the three tissues tested in individuals of African ancestry (*P* < 0.05). None of the associations met the across-tissue Bonferroni-corrected *P* value (0.0167). Cases were required to have two instances of the “Heart failure with reduced EF [Systolic or combined heart failure]” phecode, controls had no reports of “Heart failure with reduced EF [systolic or combined heart failure]” in their records. Individuals with similar codes were excluded from the analysis (congestive heart failure; nonhypertensive, congestive heart failure (CHF) NOS, Heart failure NOS, heart failure with preserved EF [diastolic heart failure], ill-defined descriptions and complications of heart disease, heart transplant/surgery, abnormal function study of cardiovascular system, symptoms involving cardiovascular system, cardiac complications, not elsewhere classified). Covariates included sex, age, median age of medical record, genotype batch, and genetic ancestry (*principal components 1*–*10*). Together, this suggests that the MICOS complex may be linked to genetic factors of heart failure, and plays a role in modulating membrane potential and oxidative stress in iPCS-CM.

## DISCUSSION

Heart failure may be intrinsically linked to mitochondria (2, 33, 61), and understanding the interplay of cristae dynamics, MICOS complex component expression, and mitochondrial dynamics during aging may aid in the development of future therapies to restore energetic production. Here, we demonstrated abnormal mitochondrial structure and impaired function related to the loss of the MICOS complex. To our knowledge, this study is the first to show that abnormal MICOS complex alters mitochondrial morphology in cardiac tissue assessed by 3-D-EM. Previous studies looking at mitochondria in aged cardiomyocytes did not find significant changes in mitochondrial number, however, there was no comprehensive quantitative analysis of the mitochondrial size and morphology (62). With the innovative 3-D-EM technology, we reported decreased mitochondrial volume and altered morphology with age. Importantly, our study combined TEM and SBF-SEM to create novel 3-D reconstructions of aging cardiac muscle cells. Although the high resolution of TEM allows us to measure cristae (42), it only creates 2-D images that do not provide accurate spatial resolutions. Therefore, the 3-D reconstructions of mitochondria by SBF-SEM allow us to better understand mitochondrial morphology. Future studies are needed that use focused ion beam-scanning electron microscopy (FIB-SEM) and thus, allow for better resolution of the details of smaller subcellular objects, such as cristae (36, 63). In the past, FIB-SEM was successfully performed in mouse cardiac muscle to characterize mitochondria, but not suborganellar structures, such as cristae (62, 64). However, mixed-microscope approaches may still be required to achieve the resolution and sample size to analyze changes in mitochondria associated with aging and organ, particularly muscle, behavior.

Previous studies have quantified mitochondria with SBF-SEM in human and mouse muscles, yet the aging heart remains understudied with 3-D techniques(36). Given that cardiac muscle is responsible for proper heart contractions, there remains a gap in our understanding of the changes of cardiac mitochondrial 3-D structure in aging. When only examining TEM, our results show that much of the age-dependent changes in mitochondrial architecture occur only from 3 mo to 1 yr, which is equivalent from adolescence to adult. However, when switching to 3-D reconstruction, we observe that mitochondrial size and morphology progressively decrease across the aging process, including from 1 to 2 yr, which better emulates geriatric aging. Interestingly, however, mitochondrial networking does not appear as affected by the aging process past 1 yr. In tandem, our 3-D reconstruction elucidates how cardiac muscle mitochondria change across aging in mice, highlighting the importance of the MICOS complex in aging and its effects on cristae morphology and mitochondrial dynamics. Future studies are needed that examine mtDNA and other modulators of cristae and mitochondrial dynamics (65) as they may provide a further understanding of the MICOS complex in relation to mitochondrial structure.

When viewing mitochondrial 3-D structure across aging, we looked for the presence of nanotunnels. Previous studies suggest that the formation of nanotunnels occurs during mitochondrial stress. Nanotunnels are mitochondrial structures that allow for the transport of materials and intermitochondrial communication (35). Nanotunnels have also been found in human skeletal muscles and in diseased states (35). We did not find evidence of nanotunnel formation in aged heart. This warrants future studies to investigate the factors leading to nanotunnel formation and whether this mechanism also exists in cardiac muscle (66).

**Figure 7.**
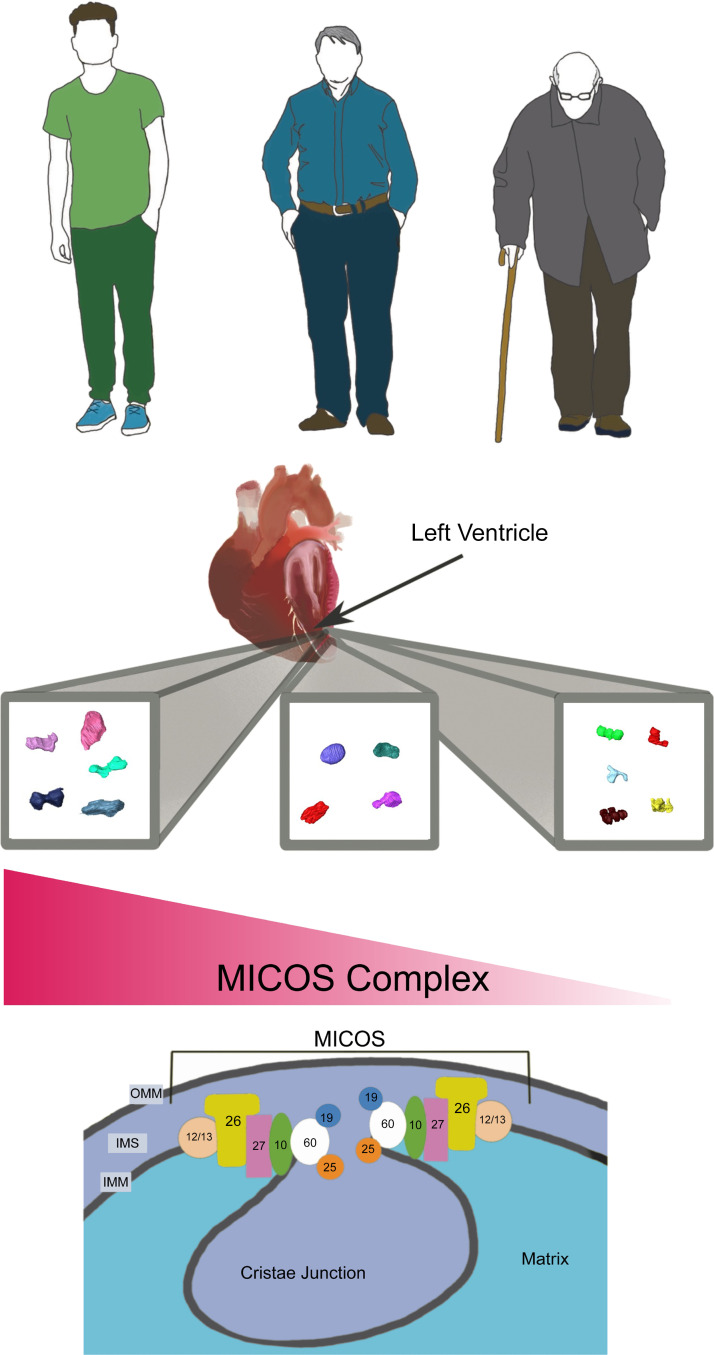
Graphical representation of loss of mitochondrial contact site and cristae organizing system (MICOS) complex across aging that may occur, affecting the structure of mitochondria. Importantly, here we show this in a murine model, which may not translate to a human model (see *Limitations*), but these findings suggest an important relation between the MICOS complex and aging which may be reproduced in human models. OMM, outer membrane; IMS, intermembrane space; IMM, inner membrane.

We observed a decline in MICOS components gene expression with age (Fig. 7). Additionally, with 3-D reconstructions, we observed that the loss of *Opa1, Mitofilin, Chchd3,* and *Chchd6* in cardiac fibroblasts may be correlated with high mitochondrial dysfunction. This was marked by smaller mitochondria, altered morphology, and reduced OCR. Given the higher percentage of fragmented mitochondria and reduced mitochondrial volume of *Opa1*-KO, *Chchd3*-KO, *Chchd6*-KO, and *Mitofilin*-KO. The exact cause of this fragmentation is unclear, although past studies have linked cristae dysfunction and mitochondrial fragmentation, potentially through mtDNA mutations (25). However, further study is needed to determine if the loss of the MICOS complex is causing mitochondrial stress-related fragmentation, modulation of fission-fusion dynamics, or acts on mitochondrial dynamics in an altogether different way.

It was predicted that these phenotypes would also arise in aging as expression of *Opa1*, *Mitofilin*, *Chchd3*, and *Chchd6* is reduced across aging (Fig. 4). In cardiac tissue, we observed that the mitochondrial number increased with age, while the area decreased (Fig. 1, *B–D*). It is possible that fission rates may be increasing over time, because of pathway changes such as *Opa1* loss or *Drp1* upregulation (5, 20). Furthermore, the loss of cristae morphology supports the occurrence of age-associated decline of the MICOS complex. When looking at 3-D reconstruction, we principally noticed alterations in mitochondrial volume across aging which parallel findings from the loss of MICOS complex. In combination, these findings suggest that the loss of MICOS complex genes may be associated with a loss of overall mitochondrial volume. However, in comparing morphological changes in aged samples with MICOS complex KO fibroblasts, we noticed a differential change in morphology. Mitochondrial morphology in the heart did not change as drastically as anticipated in response to aging. In general, the sphericity of mitochondria did not undergo significant changes (Fig. 3*I*).

Changes in mitochondrial shape are implicated in heart failure (33), which makes the specific study of cardiac muscle across aging essential for the development of new therapeutics. This study showed less networking, as measured through MBI, in cardiac mitochondria across aging than other studies have suggested (Fig. 3). It is possible that some cardiac metabolic pathways are more resilient to aging changes, supporting the retention of mitochondrial morphology. Interestingly, a recent study showed that spermidine treatment restores mitochondrial morphology in the aged hearts (62), potentially indicating that spermidine differentially is regulated specifically in cardiac tissue. However, future studies are warranted to examine age-dependent mitochondrial changes in other tissues, to better understand tissue-specific mitochondrial changes that occur during aging.

Looking at iPCS-CMs, the loss of the MICOS complex led to increased ROS (Fig. 6), consistent with previous studies in other models showing that loss of the MICOS complex may cause oxidative stress (67). Although the mitochondrial free radical theory of aging has been proposed for decades (68), the detailed mechanistic interplay between ROS and mitochondrial dynamics remains incompletely understood. Previous studies suggest that a positive feed loop within mitochondria promotes more ROS and thus, amplifies the ROS-related damage and structural decline (69). Consistent with this, loss of *Opa1* has been reported to cause the accumulation of ROS (70). Taken together, it is possible that during the aging process, the loss of *Opa1* and the MICOS complex allows for more oxidative stress to occur, resulting in ROS-induced ROS and the age-related functional decline of mitochondria (71). TMRE we used here to validate TEM findings that there are no significant changes in active mitochondria, thus oxidative stress changes can be mainly attributed to altered generation (72). Interestingly, we also noticed that when using TMRE as a baseline measurement of mitochondrial membrane potential, we diverge from the literature in showing there is no change in the membrane potential. Loss of the MICOS complex, specifically Mic60, results in a decrease in membrane potential in Drosophila (56). A loss of membrane potential in OPA1 loss has also been seen in a murine model (14). Yet, TMRE may not be the best monitor of membrane potential as loss of negative charge affects its retention; thus, future studies may better investigate the MICOS-dependent loss of membrane potential (72, 73). Using the BioVU BioBank, we also observed linkages of *Chchd6* to factors of heart failure, with a differential ethnicity association (Supplemental Fig. S4), suggesting that loss of the MICOS complex and concomitant modulation of mitochondria structure has important implications in human health which must need to be further elucidated.

In summary, we combined TEM and 3-D reconstructions to evaluate mitochondria and cristae morphology in male murine cardiac muscle. Our findings indicate that the MICOS complex decreases with age, and loss of the MICOS complex results in mitochondrial dysfunction and changes in mitochondrial structure. These findings better our understanding of how mitochondrial structure quantitively changes in cardiac muscle, and aid in understanding a potential that can be targeted to protect cardiac muscle mitochondria from complete fragmentation. Future studies are needed to explore this mechanism and continue to elucidate the link between age-related changes in cardiac cristae structural integrity, the MICOS complex, mitochondrial dysfunction, and ROS.

### Limitations

While we explored mitochondrial structure of around 750 mitochondria in each age cohort of male murine myocardium tissue, there are several potential limitations of our study. While the overall mitochondrial count was high, the quantity of mice in each age cohort is limited, although we did not note large heterogeneity between the mice for most mitochondrial quantifications. Notably, while Fig. 7 shows a human model, this study was performed in a male murine model. There are several known critical differences between human and rodent hearts, which have differential developmental processes although they are generally considered to have similar anatomy (74). For example, while human hearts are predominantly composed of β-myosin heavy chain (MHC), in murine hearts, α-MHC is relatively more abundant while pathophysiology and aging cause a shift toward β-MHC (75, 76). Notably, myocardial performance is partially dependent on the relative quantities of these MHCs, suggesting that they may modulate mitochondrial structure and function (77). While mitochondrial-related cardiovascular pathophysiology parallels each other between these models, greater research is still necessary in potential model-dependent differences. Beyond this, while we show through TEM that there are no sex-related differences on mitochondria across aging (Fig. 1), future studies may still consider performing 3-D reconstruction of female mice. While it is commonly understood that men are more vulnerable to age-related cardiovascular pathologies, female hearts have been shown to have increased resilience toward oxidative stress (78), which suggests that there may be a sex-dependent response to ROS. Finally, while the study sheds light on some of the molecular mechanisms underlying age-related decline in mitochondrial function, it does not address potential interventions or treatments to prevent or reverse this decline. Future research may look at whether restoring the MICOS complex can restore these age-related changes. Since we only show a loss of MICOS transcripts over aging, it remains to be elucidated if increasing MICOS protein amount may restore mitochondrial structure.

## DATA AVAILABILITY

Data will be made available upon reasonable request.

## SUPPLEMENTAL DATA

10.6084/m9.figshare.22861616.v3 Supplemental Figs. S1–S4 and Videos S1–S6: https://doi.org/10.6084/m9.figshare.22861616.v3.

## GRANTS

This work was supported by National Institute of Health (NIH) Grants T-32 DK007563 (multidisciplinary training in molecular endocrinology to Z.V. and A.C.) and T32 DK101003 (integrated training in engineering and diabetes) (to D.C.S.); Burroughs Wellcome Fund (BWF) Postdoctoral Enrichment Program (PDEP) Grant 1022355 (to D.C.S.); United Negro College Fund/Bristol-Myers Squibb (UNCF/BMS)-E.E. Just Postgraduate Fellowship in Life Sciences Fellowship and BWF/PDEP Grant 1022376 (to H.K.B.); National Science Foundation (NSF) Grant MCB 2011577I (to S.A.M.); U.S. Dept. of Veterans Affairs Office Research Grant I01 BX005352 and NIH Grant R01HD090061 (to J. A. Gaddy); NSF Grants EES2112556, EES1817282, and MCB1955975 and CZI Science Diversity Leadership Grant 2022–253614 (from the Chan Zuckerberg Initiative DAF, an advised fund of Silicon Valley Community Foundation to S.M.D.); and UNCF/Bristol-Myers Squibb E.E. Just Faculty Fund, Career Award at the Scientific Interface (CASI Award) from BWF, BWF Ad-hoc Award, NIH Small Research Pilot Subaward to 5R25HL106365-12 from the National Institutes of Health PRIDE Program, DK020593, Vanderbilt Diabetes and Research Training Center for DRTC Alzheimer’s Disease Pilot & Feasibility Program; and CZI Science Diversity Leadership Grant 2022-253529 (from the Chan Zuckerberg Initiative DAF, an advised fund of Silicon Valley Community Foundation to A.H.J.).

## DISCLAIMERS

The content is solely the responsibility of the authors and does not necessarily represent the official views of the NIH. The funders had no role in study design, data collection and analysis, decision to publish, or preparation of the manuscript.

## DISCLOSURES

No conflicts of interest, financial or otherwise, are declared by the authors.

## AUTHOR CONTRIBUTIONS

V.E., D.-F.D., and A.H.J. conceived and designed research; Z.V., K.N., L.V., E.G.-L., T.A.C., J.S., J.L., B.K., S.B., A. Koh, T.M.-F., K.V.A., E.Z., N.D., and A.H.J. performed experiments; Z.V., K.N., L.V., E.G.-L., T.A.C., J.S., J.L., B.K., S.B., T.M.-F., K.V.A., N.D., and A.H.J. analyzed data; Z.V., K.N., L.V., E.G.-L., T.A.C., J.S., J.L., D.-F.D., and A.H.J. interpreted results of experiments; Z.V., K.N., L.V., E.G.-L., H.K.B., A.G.M., and A.H.J. prepared figures; Z.V., K.N., L.V., H.K.B., A.G.M., A.C., J.A., B. Rodriguez, T.B., B.S., D.C.S., K.K., H.-J.K., C.S.E., B.T., A.K.R., S.A.M., B.C.M., S.M.D., J.A. Gaddy, B. Riggs, J.A. Gomez, M.A.P., V.E., D.-F.D., and A.H.J. drafted manuscript; Z.V., K.N., L.V., T.A.C., H.K.B., A.G.M., A.C., J.A., B. Rodriguez, T.B., B.S., D.C.S., K.K., H.-J.K., C.S.E., B.T., A.K.R., E.Z., S.A.M., B.C.M., S.M.D., J.A. Gaddy, B. Riggs, C.W., M.M., J.A. Gomez, M.A.P., V.E., D.-F.D., and A.H.J. edited and revised manuscript; Z.V., K.N., L.V., E.G.-L., T.A.C., J.S., J.L., H.K.B., A.G.M., A.C., J.A., B. Rodriguez, B.K., S.B., T.B., B.S., D.C.S., K.K., H.-J.K., A. Koh, C.S.E., B.T., A.K.R., T.M.-F., K.V.A., E.Z., N.D., S.A.M., B.C.M., S.M.D., J.A. Gaddy, B. Riggs, C.W., A. Kirabo, M.M., J.A. Gomez, M.A.P., V.E., D.-F.D., and A.H.J. approved final version of manuscript.
